# The Close of the Year

**Published:** 1872-12

**Authors:** 


### THE CLOSE OF THE YEAR.

At the close of the year, we take occasion to thank our friends for their generous support. The COMPANION completes its second volume, and with a copious index, is now ready for the binder. During the past year we have been encouraged and cheered by an host of well-wishers, while the steady increase of the circulation of our journal has placed it among the foremost in the Union. We are truly thankful for the kind appreciation of our labors so evidently given by our brethren of every section of the country.

Many friends have aided us, by pen and by personal effort, in establishing the COMPANION upon a firm basis. To these, one and all, we tender our heartfelt gratitude. We shall never forget them.

The COMPANION now enters upon its Third Volume—a success over all difficulties. During the coming year it will, by the blessing of Providence, labor, as in the past, for the glorious objects for which it was inaugurated. It will endeavor to befriend the man of science, and monthly unfold to the practical, busy physician, the latest and most approved discoveries in practical medicine, and thus become a faithful adviser at the bed-side.

Under our new arrangements, the publisher will be alone responsible for the mechanical execution and the punctual publication of the COMPANION. As before stated, the business department will be under the control of our Business Manager—Col. CHARLES WHITEHEAD, a cultivated gentleman—who will give his personal attention to that department, and see to the mailing of the journal. All complaints, heretofore made, in consequence of irregularity of transmission and other matters pertaining alone to the business department, will now, we believe, under his management, cease, as he will devote his entire time to the business. All communications of a business character, should be addressed to him, and they will receive prompt attention.

The Editors pledge themselves to do their best in gathering, from every quarter, the best *practical* medical literature. Their aim will be to cull the practical, and in this way to strengthen the hands of the busy practitioner, who demands the known facts of medical science and its therapeutics.

